# Knowledge of symptoms and self-management of hypoglycaemia amongst patients attending a diabetic clinic at a regional hospital in KwaZulu-Natal

**DOI:** 10.4102/phcfm.v8i1.906

**Published:** 2016-06-17

**Authors:** Anthony Ejegi, Andrew John Ross, Keshena Naidoo

**Affiliations:** 1 Department of Family Medicine, Ngwelezana Hospital, South Africa; 2 Department of Family Medicine, University of KwaZulu-Natal, South Africa

## Abstract

**Background:**

Diabetic patients on insulin and sulphonylureas are at risk of developing hypoglycaemia. Many patients do not respond appropriately because of poor knowledge and understanding of the symptoms of hypoglycaemia, which if not promptly treated can lead to permanent neurological and renal damage. Hypoglycaemic complications can be avoided if patients have a good knowledge of the early symptoms of hypoglycaemia and know how to respond appropriately.

**Aim:**

The aim of this study was to assess the knowledge of adult diabetic patients attending a diabetic clinic about symptoms of hypoglycaemia and how they responded to these symptoms.

**Setting:**

A hospital-based diabetic clinic in northern KwaZulu-Natal.

**Methods:**

This was a cross-sectional, descriptive study involving 200 diabetic patients. Demographic data and details of current medication, knowledge of hypoglycaemia and how patients responded to the symptoms were collected using a validated questionnaire.

**Results:**

The majority of the patients had fair to good knowledge of hypoglycaemia; however, less than 25% knew what action to take when they experienced symptoms suggestive of hypoglycaemia.

**Conclusion:**

There is a need to improve the education given to diabetic patients on stepwise measures to take to avoid life-threatening complications associated with hypoglycaemia.

## Introduction

Diabetes mellitus (DM) is the most common non-communicable disease in the world, with the largest number of DM patients being in developing countries.^1^ An estimated 387 million people worldwide have DM, most of whom are aged between 40 and 59 years, with the World Health Organization predicting that by 2025 this number will have risen to 592 million adults globally.^1^

DM is characterised by prolonged periods of persistently high blood glucose levels and is broadly divided into type 1 DM (also known as juvenile-onset diabetes or insulin-dependent DM) and type 2 DM (also known as maturity-onset or non-insulin-dependent DM). Approximately 90% of DM patients have type 2 DM.^2^

As part of diabetic management, glycaemic goals must be set appropriately. The 2012 Society for Endocrinology, Metabolism and Diabetes of South Africa (SEMDSA) guidelines recommend target levels of HBA1C ranging from 6.5% to 7.5% and allow elderly patients to have a HBA1C level as high as 8%. Achieving glycaemic targets has been shown to reduce macrovascular and microvascular complications associated with prolonged hyperglycaemia. Although there is evidence that tight control of blood glucose leads to a greater reduction in micro- and macrovascular complications,^3^ too tight control of hyperglycaemia may lead to hypoglycaemic complications which are considered more dangerous in most DM patients than the complications associated with hyperglycaemia.^4^

Achieving these glycaemic targets involves lifestyle changes, which may include weight management, exercise and dietary changes. In addition, most patients require pharmacological agents to achieve optimum glycaemic control. The choice of pharmacological agent must be individualised, based on age, pre-existing risk factors for hypoglycaemia such as renal and liver problems, type of DM and the concomitant use of other medications.

All type 1 DM patients require insulin and those with type 2 DM are initially started on metformin, to which a sulphonylurea is added if the blood sugar levels remain above target levels. Long-acting insulin given at night is initially added to the treatment of type 2 DM patients who do not reach glycaemic targets despite maximal oral medication.^3,4^ Many patients with type 2 DM are eventually switched from oral medication to biphasic insulin.^3,4^

An integral part of the management of DM is appropriate diabetes education, which should include information about adherence, weight management, diet (including alcohol intake) and exercise as well as information on what constitutes hypoglycaemia and how it can be prevented and treated.^5^ The United Kingdom Prospective Diabetes Study (UKPDS) group trial involving 5100 newly diagnosed type 2 DM patients found that amongst diabetic patients aged 25–65 years, 2.5% reported substantive hypoglycaemic events (grades 2–4) each year, and 0.55% had experienced a major hypoglycaemic episode which was life-threatening (grade 3 or 4).^6^ A study done in Ninewell in the United Kingdom in 2010, involving 8655 diabetic patients, showed that 7.8% of those on insulin and 0.8% of those on sulphonylureas had presented to emergency facilities with life-threatening symptoms of hypoglycaemia.^7^ These episodes were often because of poor knowledge and understanding of hypoglycaemia, inability to recognise the symptoms of hypoglycaemia and a lack of knowledge about how to respond appropriately.^8^

Hypoglycaemia is diagnosed when the blood glucose level falls below 4 mmol/L.^3^ However, symptoms may occur at higher levels when glucose levels drop rapidly. Rapid treatment of hypoglycaemia is essential as it can cause major complications in the brain, kidneys and other vital organs, and can lead to permanent neurological and renal damage.^9^ Hypoglycaemia is managed with either oral or intravenous glucose and/or intramuscular glucagon.^10^ To avoid the serious complications associated with hypoglycaemia, it is important for all patients on insulin and sulphonylureas to recognise the symptoms of hypoglycaemia and to be able to respond to them immediately.^11^ In addition, all patients on insulin and sulphonlyureas should ideally be able to self-monitor their blood glucose and to adjust their diet or medication as necessary.^12^

The international literature suggests that hypoglycaemia is poorly recognised by many at-risk diabetic patients on insulin and/or sulphonylureas.^6,7,8,13^ A national online survey in 2011 amongst 2530 adult patients with type 2 DM done in San Diego in California in the United States showed that many patients were unable to list the leading causes of hypoglycaemia; 27% of patients did not know that skipping meals could cause hypoglycaemia and 35% did not know that some diabetic medications could increase the risk of hypoglycaemia. Forty-six per cent of the participants did not know that exercise might bring on hypoglycaemia, particularly when combined with some medications.^13^

Currently, there are no South African data on the knowledge of patients that attend public sector DM clinics about hypoglycaemia and their response to these symptoms. The purpose of this study was to fill this gap and to assess the knowledge of adult DM patients about symptoms of hypoglycaemia and how they responded to these symptoms, particularly early symptoms, in order to ensure early and appropriate management.

Ethical permission to carry out this study was given by the Biomedical Research Ethics Committee of the University of KwaZulu-Natal (Ref. BE326/13). Permission to conduct this study was also obtained from the KwaZulu-Natal Department of Health and the hospital management. All patients signed informed consent after reading the information sheet provided.

## Research methods and design

### Study design

This was an observational, cross-sectional and descriptive study.

### Setting

The Family Medicine Department is responsible for running a weekly outpatient diabetic clinic at a 560-bed regional hospital in northern KwaZulu-Natal. Patients who have multiple co-morbidities, and those for whom it is difficult to achieve diabetic control, are referred to the diabetic clinic from the primary health care clinics which are supported by the hospital. Approximately 50–60 patients are seen at the diabetic clinic each week, with up to 50% of the patients being new referrals. In total approximately 1000 patients are seen at the diabetic clinic annually. Patients are seen monthly until stable and then where appropriate patients are referred back to their Primary Health Care (PHC) clinic.

### Sample size and sampling

A sample size of 200 patients, representing 20% of the patients seen in a year, was considered sufficient for a small descriptive study.^14^ Inclusion criteria were all adult patients aged over 18 years attending the diabetic clinic who were willing to participate in the study. Exclusion criteria included pregnant women and those aged below 18 years. All patients who met the inclusion criteria presenting during the months of May–June 2014 were asked by a trained assistant to participate in the study, until a total of 200 patients was reached. All patients approached agreed to participate and gave oral or signed informed consent. Illiterate patients gave verbal consent and were helped by the research assistant to complete the questionnaire.

### Data collection

Data were collected using a previously validated questionnaire from a study done among diabetic patients in India in 2008.^15^ The questionnaire asked for demographic information, duration of diabetes, current medication, self-monitoring practices, knowledge of the symptoms and their responses to hypoglycaemic symptoms. The questionnaire was available in either English or isiZulu which is the language of the majority of patients who attend the diabetic clinic at this regional hospital. To start with, a pilot study was conducted by the researcher and research assistant involving six patients chosen conveniently at the diabetic clinic to ensure that patients were able to complete the questionnaire satisfactorily in isiZulu. Analysis of these questionnaires showed that participants were able to complete the questionnaire.

The questionnaire took on average 10 minutes to complete and participants were asked to complete the questionnaire on their own where possible while waiting to see the doctor. Charts of those patients who completed the questionnaire were marked to ensure that patients did not participate more than once.

### Data analysis

A scoring system, previously used and validated in a study carried out in India in 2008,^15^ was used to assess the knowledge of hypoglycaemic symptoms and how patients responded to them. Patients were asked to list what they would feel if their blood sugar was low. Patients were rated as having ‘poor knowledge’ if they were unable to list any symptom, ‘fair’ if they listed up to two symptoms, ‘good’ if they were able to list between three and six symptoms and ‘very good’ if they listed more than six symptoms. Patients were also asked what they would do if their blood sugar level was low. Their action was scored as ‘good’ if they listed use of sugar solution or ingesting sweets or immediate consultation with their healthcare provider. Their action was assessed as ‘poor’ if they mentioned that they did not know what to do or that they would drink water or any other unsweetened beverage. All data that were collected were entered into the Statistical Package of Social Sciences (SPSS) for analysis. Association between age group and knowledge of hypoglycaemia as well as between level of education and knowledge of hypoglycaemia were assessed using the Pearson Chi- squared test. A *p*-value of < 0.05 was taken as significant.

## Results

A total of 200 diabetic patients were interviewed, of whom 152 (76%) were women and 48 (24%) were men. The majority of the participants 132 (69%) were from the district serviced by the regional hospital. Twenty three (11.5%) were aged between 18 and 39 years, and more than half (55%) were aged between 40 and 59 years (see Table 1). The median age was 54.5 years. Thirty patients (15%) were only on metformin; 40 (20%) were on metformin and a sulphonylurea; 98 (49%) were on metformin, insulin and sulphonylurea; 13 (6.5%) were on metformin and insulin; and 19 (9.5%) were only on insulin (Table 1).

**TABLE 1:** Age of participants against profile of medication use (*N* = 200).

Medication	Aged 18–39 years (%)	Aged 40–59 years (%)	Aged above 60 years (%)	Total no. of participants (%)	%
Metformin only	5 (16.7)	12 (40)	13 (43.3)	30	15
Metformin and sulphonylurea	0 (0.0)	30 (75)	10 (25.0)	40	20
Metformin, sulphonylurea and insulin	3 (3.1)	55 (56.1)	40 (40.8)	98	49
Metformin and insulin	0 (0.0)	13 (100)	0	13	6.5
Insulin only	15 (78.9)	0	4 (21.1)	19	9.5

**Total**	**23**	**110**	**67**	**200**	**100**

With regard to knowledge of the symptoms of hypoglycaemia, 66% (100/200, 50% good; 32/200, 16% very good) of patients had good or very good knowledge, whilst only 8% of patients were unable to identify any symptoms of hypoglycaemia. However, less than a quarter of patients (48/200, 24%) were aware of what to do when experiencing symptoms of hypoglycaemia.

Only 26 patients (13%) had no education; most participants had completed primary school and eight (4%) had university-level education (see Table 2).

**TABLE 2:** Comparison of knowledge of symptoms and age and level of education (*N* = 200).

Variables	Knowledge of symptoms				Total	P*

	Poor	Fair	Good	Very good		
**Age (years)**						
18–39	2 (8.8%)	9 (39.1%)	12 (52.1%)	0 (0%)	23	0.048
> 40	14 (7.9%)	43 (24.3%)	88 (49.7%)	32 (18.1%)	177	

**Total**	**16**	**52**	**100**	**32**	**200**	

**Level of education**						
No education	9 (36%)	1 (4%)	10 (40%)	5 (20%)	25	< 0.001
Primary	4 (4.8%)	23 (27.7%)	44 (53.0%)	12 (14.5%)	83	
High school	3 (3.6%)	25 (30.1%)	42 (50.6%)	13 (15.7%)	83	
University	0 (0%)	3 (33.3%)	4 (44.5%)	2 (22.2%)	9	

**Total**	**16**	**52**	**100**	**32**	**200**	

* Pearson chi-squared test.

There was no statistical association between being on insulin and knowledge of hypoglycaemia or how to manage hypoglycaemia. There was also no statistical association between good and excellent knowledge of the symptoms of hypoglycaemia and the ability to manage hypoglycaemia.

Forty-eight patients (24%) had a glucometer at home, whilst 68 (34%) were able to check their sugar levels at home or at a nearby pharmacy. Eighty-six patients (43%) indicated that they adjusted their medication at home.

## Discussion

Two hundred adult diabetic patients participated in this study, the majority of whom (88.5%) were older than 40 years and had type 2 DM. This is in keeping with international literature, which suggests that approximately 90% of all diabetic patients fall into the type 2 DM category.^1^ These findings are also consistent with a study in India in 2013, which found that most of the patients presenting for care at a diabetic clinic in an urban hospital were aged above 40 years, and over 90% had type 2 DM.^16^ Worldwide the ratio of women to men with DM is 2:1.^2^ In this study, 76% of the patients were women, which is consistent with other studies which have demonstrated a greater number of women accessing medical treatment for type 2 DM at an urban hospital.^16^ This suggests greater health-seeking behaviour amongst women with regard to type 2 DM or the inaccessibility of healthcare services providing care for men with type 2 DM, and needs further study. Although just less than 25% of the patients in this study were men, it is encouraging that more men were able to access medical treatment than in the study done in India where only 23.5% of the study population were men.^16^

The majority of patients (170; 85%) were on sulphonylureas or on insulin (Table 2) and therefore at risk of developing hypoglycaemia. In Britain, in 2005, it was estimated that the annual risk for hypoglycaemia associated with use of sulfonylureas was 1.8% per person per year. This risk is age dependent, with those older than 65 years having an annual risk of 2.0%, whilst those younger than this have a 1.4% risk of developing hypoglycaemia.^6^ The UKPDS reported on hypoglycaemic episodes over a 10-year period; insulin use was associated with a 36.5% incidence of hypoglycaemia, whilst other drugs such as chlorpropamide (a first-generation sulphonylurea) was associated with an 11.0% incidence, and glibenclamide (a second-generation sulphonylurea) was associated with a 17% incidence.^14^ A study done in India in 2008 showed that hypoglycaemia affected approximately one out of every four people with DM,^15^ whilst a community survey in San Diego in 2011 of 2530 adult patients with type 2 DM revealed that 55% had experienced at least one episode of hypoglycaemia.^13^

While this study did not specifically ask patients whether or not they had experienced an episode of hypoglycaemia, evidence from international studies would suggest that at least one in four patients would have experienced an episode of hypoglycaemia each year.^15^

It was encouraging that 132 (66%) of 200 patients reported good to very good knowledge about the symptoms of hypoglycaemia, suggesting that the education they received on diabetes is effective in covering this aspect of diabetic care. This finding is similar to that from an online survey in San Diego (2011), wherein most type 2 DM patients were aware of the symptoms of hypoglycaemia.^13^ The finding in this study is also similar to that of a similar study done in India in 2013, where two-thirds of patients had good knowledge of the symptoms of hypoglycaemia.^16^ The finding in this study is encouraging, as the study in India was carried out in a teaching hospital endocrinology clinic where the health education may have been much more focused and intense than the health education provided in the diabetic clinic in the regional hospital in KwaZulu-Natal. A study in Kaduna, Nigeria, involving 347 patients attending two different outpatient clinics (117 of whom were diabetic) showed that a smaller number (34.2%) of patients were aware of the symptoms of hypoglycaemia.^17^

However, it was of concern that 16 patients (8%) in this study were unable to list any symptoms of hypoglycaemia. This finding is however not completely unexpected, as other studies have shown that a significant number of patients on drugs which put them at risk of hypoglycaemia are unable to recognise symptoms suggestive of hypoglycaemia, which puts them at risk of severe neurological impairment if the unrecognised symptoms result in delay in the management of hypoglycaemia.^17^

In this study, there was a statistically significant association between level of education and knowledge of hypoglycaemia. Most patients who had good and fair knowledge had at least high school education. This is consistent with a study done in Saudi Arabia involving 1039 diabetic patients, which showed an association between levels of education and knowledge of hypoglycaemia.^18^ There was also a statistically significant association between age and fair to good knowledge of the symptoms of hypoglycaemia. This may be because of ongoing health education given to patients who attend the diabetic clinic over a prolonged period of time; however, further research is needed to explore this in greater detail.

It was of concern that despite 184 (92%) of the patients having at least fair knowledge of hypoglycaemia, less than a quarter knew what actions to take, suggesting a need to improve this aspect of health education in diabetes clinics. This finding is worse than that of a study in Kaduna, Nigeria, where over 50% of the diabetic patients were aware of how to manage hypoglycaemia.^17^ Diabetic education, a key aspect of diabetic management, has been shown to influence knowledge of and ability to manage hypoglycaemia.^15,19^ The fair, good and excellent knowledge of symptoms of hypoglycaemia, yet the poor knowledge of what to do probably reflects the lack of emphasis of the diabetic education given to patients. This finding is however unexpected as the SEMDSA guidelines on which the diabetes education is based has a detailed section on both the symptoms and management of hypoglycaemia.^3^ Further research is needed into what is covered in the diabetic education sessions at the clinic, so that gaps identified (e.g., a lack of teaching about how to manage hypoglycaemia) can be incorporated into the local diabetes self-management education programme.

Sixty-eight (34%) of the patients reported their ability to self-monitor their glucose levels, even though 83 (43%) patients reported self-adjusting their medications at home. This finding is difficult to explain, as self-adjustment of medication requires a good understanding of the role that diet, physical activity and pharmacotherapy play in determining blood glucose levels. Without the ability to monitor the actual glucose level in the blood, it is impossible to effectively self-adjust medication. This worrying finding suggests that some patients are self-adjusting their medication at home based on how they feel, without being able to measure their blood sugar objectively. This may put them at risk of developing hypoglycaemia or hyperglycaemia, and suggests a need for better education about diabetes and adjusting their medication, and the need to supply home glucometers to diabetic patients so that they can self-monitor and safely self-adjust their medication as appropriate.

A third of the patients (34%) self-monitoring their blood sugar level (SMBG) is consistent with a study in Japan in 1997, which showed that only 35% of patients on insulin performed SMBG.^12^ However, the SEMDSA guidelines suggest that every patient on insulin should be able to self-monitor. In many Western countries, over 80% of diabetic patients on insulin are self-monitoring.^12,19^ Although the figures for self-monitoring are lower for patients on sulphonylureas in Western countries, up to 50% of patients in these countries are able to self-monitor their glucose levels.^19^

Numerous trials have assessed the impact of SMBG on glycaemic control. Amongst patients with type 1 DM, SMBG has been associated with improved health outcomes,^20^ with a linear correlation between frequency of SMBG and reductions in HBA1C amongst such patients demonstrated in a study in Scotland.^7^ Amongst patients with type 2 DM, a higher frequency of SMBG was associated with better glycaemic control amongst those on insulin who were able to adjust their regimen.^21^ A study done in Japan in 2012 involving 137 diabetic patients showed that SMBG was beneficial for glycaemic control and was useful for those patients on oral hypoglycaemic agents.^4^ Other studies, however, have suggested that SMBG has not achieved its true potential as an aid to improving glycaemic control, particularly in type 2 DM patients.^22^ There is, however, agreement that self-monitoring is only useful if patients are well educated and have sufficient understanding of DM to be able to use the information obtained from self-monitoring to take informed decisions on adjusting dosing of insulin and oral hypoglycaemic agents.^12,19,22^

As there is evidence to suggest that self-monitoring and self-adjustment of medication improves glycaemic control and helps in the prevention of hypoglycaemia in patients with type 1 DM^3,20^ and type 2 DM,^20,22^ appropriate education and glucometers should be provided so that all patients are able to self-adjust their medication safely in response to changes in diet and physical activity. The findings from this study, however, show that most patients seen in this public sector hospital are far from this ideal scenario, and are at risk of developing hypoglycaemia or hyperglycaemia because of their inability to self-monitor their glucose levels.

The low socio-economic status of most patients accessing government services as well as the many competing demands for government resources may account for the low number of patients with home glucometers who are thus able to self-monitor their blood glucose.

### Limitations

This was a small observational study in one public hospital setting, the questionnaire was self-administered by the majority of patients and there was no opportunity to confirm the responses. For these reasons, the findings might not be applicable in other settings.

## Conclusion and recommendations

Knowledge about the symptoms of hypoglycaemia was fair to good in the majority of patients who participated in this study. However, practical knowledge of what needs to be done in the event of hypoglycaemia seems to be lacking.

There is a need to improve the education of diabetic patients on stepwise measures to be taken in order to avoid life-threatening complications associated with hypoglycaemia. In addition, there is a need for greater education about who should adjust their medication and what parameters should be taken into consideration when self-adjusting medication. Consideration should also be given to greater provision of home use glucometers as part of the patient care plan in public hospital diabetes clinics.
